# Behavioural Indicators of Pain and Suffering in Arthropods and Might Pain Bite Back?

**DOI:** 10.3390/ani13162602

**Published:** 2023-08-12

**Authors:** Robert W Elwood

**Affiliations:** School of Biological Sciences, Queen’s University Belfast, Belfast BT9 5DL, UK; r.elwood@qub.ac.uk

**Keywords:** Mandibulata, Chelicerata, nociception, pain, traumatic mating, contests, venom

## Abstract

**Simple Summary:**

Pain is an unpleasant emotional state that produces behavioural changes to minimize future tissue damage and promote recovery and survival. These behavioural changes have been demonstrated in crustaceans, insects, and, to a lesser extent, spiders. Other arthropod groups have received little attention with respect to pain. The examination of situations in which individuals might attempt to cause pain in order to manipulate others might offer new opportunities for research into pain in arthropods. For example, defensive venom, traumatic mating, and fighting might inflict pain. This might benefit the animal causing the pain and result in a cost to the animal in pain.

**Abstract:**

Pain in response to tissue damage functions to change behaviour so that further damage is minimised whereas healing and survival are promoted. This paper focuses on the behavioural criteria that match the function to ask if pain is likely in the main taxa of arthropods. There is evidence consistent with the idea of pain in crustaceans, insects and, to a lesser extent, spiders. There is little evidence of pain in millipedes, centipedes, scorpions, and horseshoe crabs but there have been few investigations of these groups. Alternative approaches in the study of pain are explored and it is suggested that studies on traumatic mating, agonistic interactions, and defensive venoms might provide clues about pain. The evolution of high cognitive ability, sensory systems, and flexible decision-making is discussed as well as how these might influence the evolution of pain-like states.

## 1. Introduction

Living arthropods comprise two major groups, the Mandibulata (including crustaceans, insects, centipedes, and millipedes) and the Chelicerata (including spiders, scorpions, and horseshoe crabs) [1]. They are named after their biting appendages, which differ in origin. The mandibulate homolog of the chelicerae is a pair of antennae. By contrast, the chelicerate homolog of the mandibles is the first pair of walking legs. 

Arthropods have a hard exoskeleton that surrounds the muscles and other soft tissue. They are typically segmented and possess jointed appendages that vary in function. The phylum arose in the Cambrian explosion circa 530–550 million years ago and evolved numerous clades, many of which died out (e.g., trilobites). The survivors, however, now comprise the most speciose animal phylum. They are found in marine, freshwater, and terrestrial habitats and are often highly mobile, including flight in some insects. Many arthropods have good visual abilities, albeit with different types of eyes. Chelicerates typically have eyes comprising a single lens and associated “retina” of light-sensitive rhabdomeres, but they often have numerous eyes. By contrast, the mandibulates typically have fewer eyes but each eye may be compound, with hundreds or thousands of ommatidia. Each ommatidium contains a lens and a cone that funnels light to a photosensitive organ. The long, thin ommatidia are bunched together, with each ommatidium pointing in a slightly different direction, which allows for good acuity, and they can detect the polarization of light. Arthropods have a plethora of other sensory modalities. For example, there is the widespread use of olfaction, exemplified by the remarkable perceptual abilities of male moths to detect potential mates over long distances via female pheromones [2]. These modalities enable arthropods to gather various sources of information to enable effective decisions that have important fitness-related consequences, such as locating food, nest sites, mates, dealing with competitors, navigation, and avoiding predators. 

Arthropods also detect noxious stimuli, such as heat, mechanical pressure, and chemicals [3,4,5]. These stimuli are detected by nociceptors that have naked nerve endings within the integument. Nociceptors are key to protecting animals from tissue damage and are found in many phyla, including annelids, molluscs, and chordates, as well as arthropods. The firing of nociceptors might initiate a nociceptive reflex that causes the swift withdrawal of the animal from the locality of the noxious stimulus. Alternatively, just the afflicted part of the body, for example, a leg or antenna, may be moved. Such observations demonstrate that the animal can perceive those stimuli and that the stimuli are aversive. 

In humans, exposure to noxious stimuli might lead to a negative affective state that we call pain. Pain is defined by the International Association for the Study of Pain as: “An unpleasant sensory and emotional experience associated with, or resembling that associated with, actual or potential tissue damage” [6]. Pain is presumed to be widespread amongst the chordates and there is evidence consistent with pain in cephalopod molluscs [7] and some arthropods [5,8,9]. However, the idea of pain in non-mammalian groups is dismissed by some, for example, Diggles [10] for crustaceans, or regarded as highly unlikely, for example, Adamo [11] for insects. A frequent argument against pain in these taxonomic groups is that their responses to noxious stimuli are merely nociceptive reflexes. A reflex would not require the involvement of the central nervous system, and without that, there would be no negative affective state. If this is true, then there would be little or no welfare concerns about suffering in arthropods. Certainly, the initial response to tissue damage might involve a reflex but that does not negate the possibility of a subsequent pain experience, as seems to occur in humans [8]. This makes assessing pain difficult because we cannot rely on immediate withdrawal reactions to suggest pain. Further, pain is a private experience, and arthropods cannot report their feelings. Thus, criteria are required that should be fulfilled for each taxonomic group before we might suggest if pain is possible; this is the broad approach taken here.

## 2. Possible Criteria for Pain

Various sets of criteria have been proposed, some of which were aimed primarily to examine vertebrates [12,13], some were created primarily with invertebrates in mind [8,9,14], and others attempted to cover all animals [15]. These sets of criteria differ with respect to which criteria are used, and there is limited agreement about which criteria are useful [9,16,17,18]. Here, it is proposed to start by asking questions about the expected function of pain and ask if this might provide guidance about which are the most suitable criteria.

Broadly speaking, the function of pain is to alter the long-term behaviour to mitigate the likely reduction in fitness caused by a wound. Thus, pain might cause the animal to promote healing and recovery by guarding or attending to the injury. Pain should enhance the ability of the animal to avoid or lessen subsequent exposure to the noxious, tissue-damaging stimuli. This might involve avoidance learning and a reduction in the willingness to take risks [15]. Anxiety, including heightened responsiveness to some stimuli coupled with risk aversion after tissue damage, might be highly beneficial, especially because wounded animals may be selectively targeted by predators [19,20]. Altered motivation regarding access to or use of resources is expected. Further, pain might cause the animal to give up important resources, but the costs of giving up a resource should be balanced against the benefit of avoiding the pain [21,22]. That is, we expect pain to have marked effects on behaviour in ways that the animal can best cope with the tissue damage and promote subsequent survival [8,12,23]. The six criteria listed below reflect these behavioural attempts to mitigate fitness loss as applied to arthropods:

(1) Swift avoidance learning (including forming preferences for analgesics or local anaesthetics);
(2) Anxiety and risk aversion;
(3) Long-term changes in behaviour not easily ascribed to associative learning;
(4) Trade-offs between avoidance of the noxious stimulus and other motivational requirements;
(5) Activities directed specifically towards the site of damage (rubbing) and reduction in the use of specific appendages (as in limping);
(6) Protection from further damage by limb autotomy.

These criteria include behavioural changes that go beyond those of nociceptive reflexes. However, other criteria have been proposed that relate to mechanisms by which these behavioural changes are achieved [15,19]. They include physiological changes, identifying brain structures that mediate behaviour change, the presence of nociceptors, and the descending control of nociceptive input. Whilst these are interesting, they are not as useful as criteria for pain as the behavioural changes they support. Some of these secondary criteria may be deduced from the primary, behavioural observations. For example, one suggested criterion is that the animal should possess brain mechanisms that allow nociceptive input to be integrated with other sensory inputs [9]. However, if an animal changes its behavioural response to the noxious stimulus depending on other motivational needs, i.e., a trade-off, then it must have the mechanism to do that [16,17]. When discussing the possibility of pain in arthropod taxa these secondary criteria will be mentioned but those relating to behaviour will be given prominence. 

The approach used here will not employ a formal scoring system like that of Crump et al. [9] and Gibbons et al. [24]. That is because there is little agreement about the weighting of scores from different criteria [17,18] or about the inclusion/exclusion of criteria [16]. Rather, a more relaxed approach will be used that notes whether there is evidence that supports each behavioural criterion. Experiments designed to test if behavioural criteria are upheld will be reviewed, as well as anecdotal evidence for and against pain in particular taxa. Other observations and phenomena will then be considered that might help to understand if pain occurs in arthropods. First, cases will be examined in which the male inflicts tissue damage to the female during mating and if that dissuades the female from mating with another male. Second, it is asked if pain might be inflicted during fights for specific resources. Third, venoms used for defence are examined and it is asked if they act against arthropods and if the defence might involve inducing pain. The aim will be to encourage broad investigations of pain that use different sources of information and suggest new approaches for pain research. Finally, because high cognitive ability has been suggested to be required for a pain experience, examples within the arthropods will be briefly reviewed. This will be viewed in an evolutionary framework to help assess which arthropod groups might benefit from the emotional experience of pain [25]. The possible role of consciousness in pain experience is beyond the scope of the present paper and good references on this are available [9]. 

## 3. Evidence

### 3.1. Mandibulata

#### 3.1.1. Crustaceans

Crustaceans show swift avoidance learning when repeatedly exposed to noxious stimuli. For example, marbled crayfish (*Procambarus virginalis*) in a T-maze initially showed a preference for an arm with blue light compared to an arm with white light. However, if the former resulted in electric shock, the preference was switched, in the short-term, after just one exposure to shock. Further, three exposures resulted in avoidance that lasted for 48 h [26]. Long-term avoidance was not observed, however, if the animal was subsequently kept at low temperatures or was given protein-formation inhibitors, suggesting that long-term memory was inhibited. Shore crabs (*Carcinus maenas*) offered a choice of two dark shelters in which to avoid bright light quickly selected one [27]. If that shelter was accompanied by electric shock, it failed to result in avoidance of that shelter in the next choice trial; rather, the crabs showed a strong preference to return to their first choice. However, in the following choice trial, those crabs that received a shock in the previous trial switched their choice to the alternative, safe shelter, thus indicating swift avoidance learning. Tests in later trials demonstrated that the crabs discriminated shelters by the direction of walking from the start point (left or right) rather than visual cues. Crabs that were turned around by 180° in subsequent tests walked to the wrong shelter [27]. However, when a different paradigm was used that involved crabs only having access to one shelter at a time over ten trials, they failed to avoid the shelter that was previously accompanied by electric shock in a final choice test when both could be accessed [28]. This is in keeping with other studies that showed that the sequential presentation of stimuli is less effective than simultaneous presentation in learning studies [29]. Nevertheless, the crabs appeared to show other forms of learning that reduced their exposure to shock because in later trials they escaped from the shock shelter significantly earlier than they did in early trials [28]. Other studies that demonstrate avoidance learning are reviewed by Crump et al. [9]. However, the author is not aware of studies on crustaceans that have tested possible preferences for analgesics or local anaesthetics when exposed to noxious stimuli.

Increased anxiety (wariness) following noxious stimuli has been demonstrated in crayfish, *Procambarus clarkii* [30]. These animals show a preference for dark environments, and when placed in a cross maze with two light and two dark arms, they spent more time in the dark than in the light arms. However, when they were given repeated electric shocks that induced escape responses, prior to being placed in the cross maze, they showed an enhanced avoidance of the light arms. This ‘anxiety’ was accompanied by higher levels of serotonin (5HT) in the brain [30,31]. Further, animals that were injected with 5HT, rather than being shocked, showed similar levels of anxiety to those that were shocked. Surprisingly, chlordiazepoxide (CDZ), a drug that reduces anxiety in humans, reduced signs of anxiety in crayfish. Aversive visual stimuli also induced anxiety in the crab, *Neohelice granulata*, and that state was blocked by a serotonin inhibitor [32]. Behaviour suggesting anxiety after receiving shocks was also noted in the amphipod *Gammarus fossarum* because the amphipods increased their time hiding in shelters and decreased their time swimming freely [33]. The fitness benefit of this anxiety was shown by increased survival in the presence of predatory fish [33]. The anxiolytic LY354740 reduced the time spent hiding in a dark shelter. It is interesting to note that all three studies showed that drugs used to treat anxiety in humans were effective in crustaceans, suggesting similar anxiety mechanisms in various taxa.

**Figure 1:**
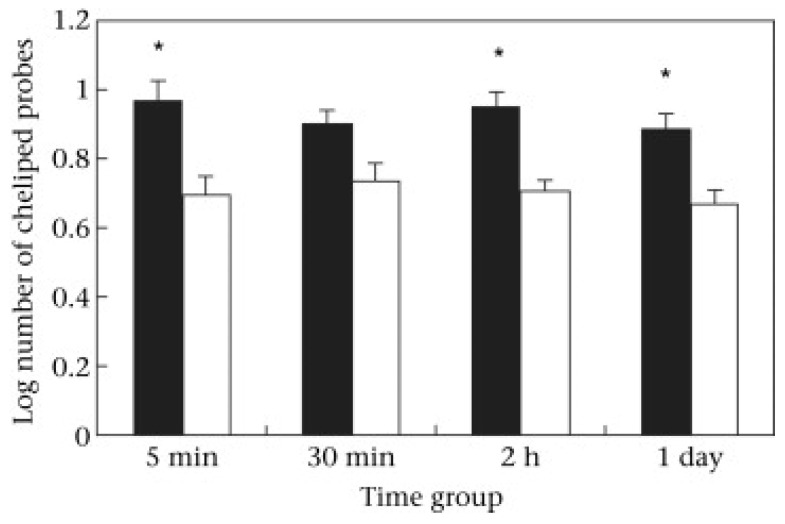
Hermit crabs that were shocked within their shells (light bars) investigated new empty shells using fewer cheliped probes before occupying those shells than did non-shocked crabs (dark bars) (F_1,112_ = 33.528, *p* < 0.0001). The times between the shock and the new shell being offered were varied and the effect of shock was significant (asterisks denote *p* < 0.05) for the 5 min, 2 h, and 1-day groups. From Appel and Elwood 2009 [36].

Long-term behavioural change caused by electric shocks on the abdomen, within the shell, has been examined in hermit crabs (*Pagurus bernhardus*) [22]. Shocked crabs and non-shocked controls were later offered a new empty shell. Those that had been shocked approached the empty shell in a shorter time and engaged in a shorter investigation prior to moving into that shell compared with non-shocked crabs. This indicated that shock within the shell caused the crab to value that shell less than did crabs that were not shocked [34,35], and were thus highly motivated to seek and take a new shell. In one experiment, the new shell was offered 20 s after the cessation of shock (or an equivalent time for the non-shocked group). A second experiment, however, used longer times between shock and the offering of a new shell [36]. This provided evidence of motivational change that lasted at least 24 h because the crabs still showed an enhanced motivation to change shells (Figure 1). 

Swift escape from noxious stimuli might appear to be a nociceptive reflex, however, it might be more complex than that [37]. This possibility was tested by assessing if trade-offs occurred between escape responses and other motivational requirements [21,22]. The rationale was that if these immediate responses to a noxious stimulus were influenced by other factors, then the animal must have integrated information from other sources with that from nociceptors, resulting in an adaptive decision rather than an inflexible reflex [9,16]. For example, some hermit crabs (*P. bernhardus*) left their gastropod shells if shocked within their shells, however, they evacuated less preferred species of shell at a lower voltage than a preferred species [21] and, if the voltage was kept constant, they were more likely to evacuate the less preferred species [22]. That is, the benefit of escape was balanced against the cost of giving up a specific resource. Further, hermit crabs were less likely to leave their shell if the odour of a predator was in the surrounding water [38]. In this case, the balance was between escaping from the noxious stimulus and the risk of predation. 

A further example of decapods modifying their escape response comes from video recordings of crayfish (*Procambarus clarkii*) when touched with a hot probe [4]. Rather than all crayfish showing a similar reflex withdrawal, some grabbed the shaft of the probe in a defensive action. That is, the response was clearly influenced by other factors and might reflect a trade-off between defence and escape [37]. These studies demonstrate that even short-term or immediate responses may not be reflexes but rather are the result of centrally organized decisions that maximize fitness (on average) following tissue damage. 

Rubbing, guarding wounds, and limping are interpreted as being consistent with pain [8,9,37]. For example, sodium hydroxide or acetic acid (both known to induce pain in mammals) applied to a single antenna of the glass prawn (*Palaemon elegans*) resulted in prolonged grooming and rubbing [3]. Grooming involved repeatedly pulling the antenna through the small chelipeds (claws) or the mouthparts, whereas rubbing was pressing and moving that antenna against the side of the tank. The responses were directed at the treated antenna significantly more than the untreated antenna, indicating an “awareness”, or at least the ability to locate the site of the noxious stimulus [37]. Further, the application of sodium hydroxide to one eye of a glass prawn caused high levels of grooming of that specific eye with either one or both first walking legs. This behaviour was not seen if just seawater was applied [8]. Similar directed activities occurred in shore crabs (*C. maenas)* when they used their claws to scratch at their mouthparts that had been brushed with acetic acid [39]. Hermit crabs that got out of their shells after being shocked on their abdomen often groomed at that site, a behaviour not seen if the crabs were removed from the shell by other means [36]. Further, edible crabs (*Cancer pagurus*) that had a clawed appendage twisted off, in the manner used in some fisheries [40], held their remaining claw over the wound during competitive interactions. Finally, when formalin was injected into a claw of a shore crab (*Hemigrapsus sanguineus*), the crab reduced the use of that appendage when walking and often pressed that claw against its carapace [41]. They also shook and rubbed the injected claw [41].

One way of mitigating tissue damage to appendages in crustaceans is to autotomise that limb. This is achieved by muscular contraction so that the limb is cast off on a fracture plane, which quickly heals. Cutting a distal joint of a claw of edible crabs (*C. pagurus*), which caused haemolymph loss, reliably induced autotomy [42]. Autotomy was also seen after claws of shore crabs (*H. sanguineus)* were injected with formalin [41] and autotomy also occurred when the walking legs of *C. maenas* were injected with acetic acid [8]. The observation of autotomy after tissue damage and loss of haemolymph might be attributed specifically to that fluid loss, however, the injection of substances known to cause pain in mammals does not result in fluid loss, yet autotomy still occurred. Further, crabs autotomized limbs when the whole animal was placed on a hot plate [43] or if the leg was subjected to electric shock [44]. These observations are consistent with the idea of pain mediating the autotomy response [8]. Further research is required, however, to determine if autotomy is a behavioural decision or simply a reflex response. That is, we need to determine if autotomy can be modified by motivational factors other than the noxious stimulus.

#### 3.1.2. Insects

Numerous studies demonstrated avoidance learning in insects and these are reviewed in detail by Gibbons et al. [24] and Pitman et al. [45]. For example, fruit flies (*Drosophila melanogaster*) avoided an odour that had been associated with electric shocks, even after just one trial, and they showed retention of the learning after 24 h [46]. Honeybees (*Apis mellifera*) associated electric shock or heat with preceding cues and similar classical conditioning has been shown in several other insects [24]. Operant conditioning to avoid noxious stimuli has also been shown, for example, in cockroaches (*Periplaneta americana*) and honeybees that learned to avoid locations in which they received electric shocks [47,48]. Other forms of learning such as trace conditioning and reversal learning probably require more complex cognitive abilities than classical or operant conditioning. In trace conditioning, there is a temporal gap between the conditioning stimulus and the noxious event, which implies a memory of the CS, and this has been shown in fruit flies [49]. Reversal learning requires the subject to learn about the switching of the predictive value of the stimuli. This could be achieved by the animal starting again in learning the task after each switch or it could learn that things can switch and thus speed up the switch in behaviour, this being demonstrated in different insects [24]. 

There has been one study on the preference for analgesics by bees (*Apis mellifera*) [50]. Injured and uninjured bees were offered a choice between a sucrose solution and sucrose with morphine, however, there was no difference in the preference for morphine even though injured bees took more food overall. Thus, there is no evidence for the self-administration of analgesics. One problem with the experiment, however, is that there is no evidence of morphine receptors in insects and thus no evidence that morphine has an analgesic effect [51]. 

One way to examine anxiety or risk aversion is to use cognitive bias tests [52]. Here, a subject receives a positive reward from one stimulus and no reward or a negative outcome with another stimulus. This may involve mixtures of two odours with a mix of 1:9 or 9:1 as the two stimuli. When this was conducted with honeybees, the bees learned which mix produced the more positive outcome. Then, some bees were shaken to induce anxiety, and their responses were compared to those of undisturbed bees. The bees were then offered ambiguous stimuli (mixtures of 3:7, 1:1, and 7:3) to determine if the bees approached these. It was shown that shaken (anxious) bees were less likely than the unshaken bees to approach these ambiguous stimuli, suggesting emotional states in these insects [52]. Similarly, bees subjected to simulated attacks by cryptic predators became more wary when foraging and lost feeding opportunities due to false alarms [53]. However, these studies did not specifically link these findings to possible pain.

Long-term behavioural change was examined in *Drosophila* that had a middle leg amputated. They subsequently showed a greater number of escape responses when placed on a surface at 38 °C than did intact animals, but only between 5 and 21 days after injury [54]. This was described as consistent with thermal allodynia, where a “painful” behavioural response is elicited by normally innocuous stimuli.

Trade-offs between avoidance of noxious heat and the requirement for quality food were examined in bumblebees (*Bombus terrestris*) [55]. Bees were allowed to forage in an arena where there was a choice between two high-quality feeders (containing a 40% sucrose solution) and two alternative feeders. The types of feeders also had colour cues. Four experimental groups of bees were each offered either a 10%, 20%, 30%, or 40% sucrose solution in the alternative feeders. The bees were allowed to forage when the feeders were all at room temperature and the bees learned to use the higher concentration feeders. Subsequently, the high-quality feeders were heated to a noxious level, but how much these were avoided depended on what was in the alternative feeders. If those feeders had low concentrations of sucrose, the avoidance of the heated feeders was lower than if the alternatives had high concentrations. That is, the bees traded off food quality with avoidance of a thermal noxious stimulus.

Directed grooming at the site of a noxious stimulus has also been reported for bumblebees (*B. terrestris*) [56]. Bees were either touched on one antenna with a heated iron or with a non-heated iron and then observed to determine which antenna (touched or untouched) and type of touching (heated or nonheated) received the most grooming. In the two minutes following the touch, there was little antennal grooming of the untouched antennae, and little directed towards the touched antenna if the iron was not heated. However, there was significantly more grooming of the specific antenna touched with the heated iron. This indicates that touching per se is not noxious but touching with a heated iron is, and the bees directed their grooming towards the site of the noxious stimulus.

Autotomy occurs in many insects, for example, field crickets (*Gryllus bimaculatus),* various leaf-footed bugs (Hemiptera: Coreidae + Alydidae) [57], and stick insects (*Didymuria violescens*) [58]. It occurs when a leg is damaged due to interspecific competition or failed predation attempts or when the leg is held [57]. Autotomy following damage is consistent with the expectation of pain. 

The idea that insects might experience pain is frequently dismissed because of observations of apparently normal behaviour continuing while the insect is being damaged. For example, male praying mantids may be eaten by their females during mating but show no attempt to escape [59]. It is argued that if the male could feel pain, then it would not continue with copulation. However, we should consider the fitness benefits to the male if he escaped and the possible loss of fertilizations from that attempted mating [60]. The male might survive but if it cannot mate successfully with another female to offset the lost fertilizations with the current female, then it might lose the chance of siring a brood. Thus, it might be beneficial in genetic terms not to struggle so that they ensure copulation continues with the successful transfer of sperm. However, males that have yet to initiate copulation with the female may struggle to escape the predatory attempts of females [60]. In this situation, the males show vigorous waving and pushing with the legs in apparent attempts to escape, which is consistent with nociception and possibly pain. The observation of some males not struggling during mating when being bitten, but others showing vigorous escape responses when attacked outside of mating, suggests that males may suppress the neural control of the response to tissue damage specifically when mating [15,51]. This modulation of responses suggests a trade-off between escape and the opportunity to mate; it deserves further investigation, as does the possibility of the descending control of nociception/pain [51].

#### 3.1.3. Centipedes and Millipedes

Centipedes are fast-moving predators whereas millipedes are slow-moving and consume mostly vegetable matter. No studies have been made that might suggest pain in these animals other than the autotomy of legs being noted in centipedes [61]. Thus, there is ample scope for research on these two groups.

### 3.2. Chelicerata

#### 3.2.1. Spiders

Several experiments are directly relevant to investigating the possibility of pain in spiders. For example, avoidance learning has been demonstrated in the wolf spider (*Schizocosa avida*) when they suffer damage when escaping from a predatory attempt by a scorpion; they subsequently avoided the odours of such scorpions [62]. Jumping spiders (*Hasarius adansoni*) also learned to avoid visual stimuli associated with high temperature [63] and electric shock [64,65]. Thus, spiders appear to learn from noxious experiences and then reduce or avoid those noxious events in the future [66] 

Leg autotomy also suggests pain in spiders [8]. For example, in *Argiope aurantia* [67], autotomy was noted when these spiders attempted to capture ambush bugs (*Phymata fasciata*), but the bug grasped a spider leg and probed a joint with its proboscis. Experimental penetration of the joint with a sterile pin did not cause autotomy, indicating that the saliva of the ambush bug likely had an effect. The venomous saliva of the bug is painful to humans, suggesting pain may play a part in autotomy [67]. When bee and wasp venom were injected into a spider leg, they induced autotomy [67]. Individual components of bee venom were then injected, some of which caused autotomy. The effective components were histamine, serotonin, phospholipase, and melittin, all of which induce pain in humans. The ineffective components were acetylcholine, bradykinin, hyaluridase, adrenaline, and dopamine. Acetylcholine and bradykinin induce pain in humans but not autotomy in spiders, and hyaluridase, adrenaline, and dopamine do not. Thus, substances that induce pain in humans are likely to induce autotomy in spiders, whereas those that do not cause pain in humans do not cause autotomy. 

#### 3.2.2. Scorpions

Reports of experiments and observations that are consistent with the idea of pain in scorpions are not common. However, avoidance learning has been noted. When giant whip scorpions, *Mastigoproctus giganteus*, were tested in a shuttle box in which electric shock could be applied to one side at a time, they learned to shuttle between compartments to avoid the electric shock [68]. This indicates the aversive nature of the shock for these animals and that they swiftly learn how to escape.

#### 3.2.3. Horseshoe Crabs

Atlantic horseshoe crabs (*Limulus polyphemus*) are used in the biomedical industry to check that injectable vaccines and medicines are safe from contamination by endotoxins. The blood from these animals is collected from wild-caught animals and the animals are subsequently released back to the wild. There is concern about the ethical aspects of this practice, but much relates to the level of mortality following bleeding and release and, hence, the sustainability of the populations [69]. Whilst there is concern about the potential for adverse welfare effects on individuals, very little is known about the potential for pain. Bleeding results in a considerable reduction in the remaining hemocyanin levels and mortality increases and activity levels decline with reduced blood [70,71]. Attempts to examine avoidance learning are limited and there is no convincing behavioural evidence that might be consistent with the idea of pain [72]. Given the high use of these animals, it is surprising that so little empirical evidence about sentience is available.

## 4. Discussion

This review shows that behavioural observations consistent with pain are not equally distributed among the various arthropod groups (Table 1). They are clustered primarily in the crustaceans and insects of Mandibulata, and to a much lesser extent in spiders of Chelicerata. They are virtually absent in millipedes and centipedes of Mandibulata and the scorpions and horseshoe crabs of Chelicerata. Hopefully, these neglected groups will soon receive attention to close these gaps in knowledge. 

For crustaceans and insects, there is evidence consistent with each of the six behavioural criteria. There are examples of avoidance learning, anxiety, and risk aversion, long-term changes in behaviour not easily ascribed to associative learning, trade-offs between avoidance of the noxious stimulus and other motivational requirements, activities directed specifically towards the site of damage (rubbing) and reduction in the use of specific appendages (as in limping), and protection from further damage by limb autotomy. However, there is no evidence that suggests preferences for analgesics or local anaesthetics. For spiders, there are examples of avoidance learning and autotomy. For centipedes, there is evidence for autotomy, and for scorpions, there is support for avoidance learning. For millipedes and horseshoe crabs, there are no studies that support any criterion. 

It should be acknowledged that finding evidence consistent with the idea of pain is not the same as proving pain [8,37]. Nevertheless, the accumulation of evidence for sentience in decapod crustaceans, as outlined by Birch et al. [14], was sufficiently compelling for legal changes to be made in the UK which now recognise the potential for pain and suffering in decapods (Animal Welfare (Sentience) Act 2022). Further, using the set of criteria of Birch et al. [14], Gibbons et al. [24] suggested that there is strong evidence of sentience in several orders of insects. However, those reviews used a mix of behavioural and neurological criteria to support their cases. They used some of the behavioural criteria used here, but they also examined the evidence regarding the presence of nociceptors, of brain regions that might integrate different sensory inputs and regions that integrate nociception and other sensory modalities and analgesic effects. As noted previously, the first three of these are demonstrations of the mechanisms that enable functionally important behavioural changes. 

Nociceptors are found in most metazoans but have been particularly well-described in insects, especially *Drosophila* [24]. Nociceptors have also been shown to occur in crustaceans [9,14]. The brains of arthropods show significant functional localization, and areas involved in the integration of different sensory modalities, including nociception, have been described [9,24,73]. For example, decapods have well-defined circuits and centres that allow for the integration of nociception with other sensory inputs and between different sensory modalities [74] and for links with learning centres [75]. These aspects are excellently reviewed by Crump et al. [9] who concluded that decapods have the neural capacity that would be expected if they experience pain. There is also extensive work on insect brains, with reference to the structure and abilities that integrate different sensory systems and integrate nociceptive input with other sensory inputs [24]. Further, the effects of local anaesthetics have been noted in crustacea [3,14] and insects [24]; however, the analgesic effects of opioids in these two groups remain in doubt [24,44]. Studies of anaesthetics and analgesics help shed light on the physiological mechanisms underpinning nociception in arthropods. All these observations are important in understanding the biology of this phylum. However, these previously suggested criteria for pain are related to the mechanisms mediating the behavioural changes that follow tissue damage, but they cannot be regarded as filling the function of pain. 

Physiological stress responses have been proposed as a criterion of pain [8,15,16] but were not used as such by Birch et al. [14]. For example, stressed crustaceans show elevated crustacean hyperglycaemic hormone (CHH) [76], whereas stressed insects show changes in biogenic amines (octopamine and dopamine), neuropeptides (allatostatin and corazonin), and metabolic hormones (adipokinetic and diuretic hormones) [77]. These systems have similarities to the cortisol of vertebrates. In decapods, CHH causes the release of glucose from stores of glycogen [78] and increases levels of lactate. Breaking a claw of edible crabs (*C. pagurus*) results in a swift increase in glucose and lactate in the haemolymph [42]. Pain is likely to cause stress so measuring physiological changes might provide a way of assessing pain.

However, there is a problem with using physiological changes to investigate pain because noxious stimuli typically result in escape responses and other vigorous activities that might cause physiological change. For example, in the study of anxiety in crayfish, the electric shocks used to stress the animals caused repeated tail-flick responses [30,31]. However, one study [79] attempted to distinguish between the direct stress of electric shock in causing elevated lactate from that caused by increased activity. Some shore crabs (*C. maenas*) received electric shock via wires attached to walking legs and others were wired in the same way but without shock. Some of those receiving shock showed increased activity such as threat responses, but most walked around the enclosure. Some of those not receiving shock remained stationary during the test but most walked around the enclosure. When lactate levels were examined in those animals that just walked about the enclosure, those that received the shock were substantially higher than those that were not shocked. Thus, the stress response was specifically due to the shock rather than the behavioural responses. This is consistent with the idea that the shock induces a pain-like state that is stressful to the crabs [79]. Although behavioural responses have been the focus of this review, physiological changes are also specific responses to noxious stimuli and modulate further behavioural responses to injury [20] and, thus, might be a reasonable criterion for pain. 

## 5. Do Animals Inflict Pain on Others to Gain an Advantage?

Should a pain system evolve, then individuals might be susceptible to being manipulated by being subjected to pain by other animals. Here, three situations are considered that might occur in arthropods. 

### 5.1. Tissue Damage Caused during Mating

There are many examples of male copulatory traits that reduce the chances of the female mating with a subsequent male [80]. These include copulatory plugs that impede a subsequent male and the production of chemicals that reduce the motivation of a female to mate or perhaps reduce her attractiveness to other males. Another mechanism involves the male causing tissue damage to the female to reduce her willingness to mate again [81]. One example is the evolution of spines on the male genitalia of bean weevils (*Callosobruchus maculatus*) that cause tissue damage to the female [82]. Females have reduced longevity due to the damage and act to minimise that damage by kicking at the male during mating attempts. The tissue damage might thus act as a “disincentive for unfaithfulness” [80]. That is, the tissue damage might change the long-term motivation of the female in a way that would be consistent with the idea of pain.

A second example relates to the mating of a wolf spider (*Pardosa pseudoannulata*) [83]. During insemination, the inner walls of the female genital tract are damaged with the sharp intromittent organ of the male. Females then show a reduced willingness to mate with a subsequent male. This might be due to chemical manipulation [80], but experiments demonstrated that the seminal fluid had only minor effects. Experimental damage by microinjection without seminal fluid, however, greatly suppressed mating [83]. This implies that the female does not require memory of mating or a male to show a reduction in the willingness to mate because with microinjection neither is involved. It does suggest, however, that tissue damage per se reduces the willingness of the female to mate. The long-term shift in motivation is a key expectation of pain. 

A third example is found in scorpions. Male scorpions use their chelae to take hold of the female’s pedipalps and attempt to position her so she can take up a spermatophore he has placed on the substrate [84]. During this process the male may sting or club the female, possibly to subdue a reluctant female. Some species show sexual dimorphism in the stinger and the components of the venom, suggesting that these might be important for male success in mating [85]. One might speculate that males might also benefit by inflicting painful stings that reduce subsequent mating by that female. 

Sometimes the conflict between the sexes might result in female countermeasures as is seen in female damselflies, *Enallagma cyathigerum*, that have a conspicuous vulvar spine which contacts with the male during copulation [86]. Males copulated for longer with non-virgin females that had the spine removed, showing that the spine reduced the duration of mating and might reduce the male’s ability to remove the sperm from a previous copulation. It was proposed that the “spine allows females to exert some control over copulation duration by producing enough “discomfort” or “pain” to males to reduce copulation duration” [86].

### 5.2. Possible Pain Inflicted to Win Fights over Resources

It has been suggested that intraspecific contests might alter the emotional state of the participants [87] and that losers show a negative affective state, which is presumably induced by the agonistic actions of the opponent. One interpretation of this effect is that the fight activities might induce pain in one or both opponents. For example, mantis shrimps, *Neogonodactylus bredini,* use their predatory appendages to strike each other during contests, often involving attempts by one to block the blows of the other with their armoured telson. Although obvious wounding was not observed, these blows are known to influence the motivation of an opponent to stay in the contest [88]. Another example of striking is seen in the shell fights of hermit crabs, which hit their shell against that of an opponent. The power of this shell rapping has a direct effect on the motivation of the opponent, and, thus, the probability of it giving up and abandoning the shell [89]. It shows similarities to the observations of hermit crabs receiving electric shocks on the abdomen, which can induce shell evacuation, consistent with ideas about pain [21,36,90]. However, decisions in fights are not just about the ability of opponents because losers typically persist for longer when they are contesting a high-value resource [91]. Arthropods (and other animals) accept higher costs in fights over high-value resources and possibly fight to the death when the resource is vital for reproduction [91]. That is, with high-value resources, we expect to see descending control of nociception (and pain) during fights, but we expect to see less descending control with lower-value resources. This would match observations about the trade-offs between pain avoidance and keeping valued resources that have been described above for crustaceans and insects. Thus, pain would provide a mechanism by which fight decisions are optimised, not only in arthropods but in a wide range of other animals. 

### 5.3. Chemical Warfare and Possible Pain

Many arthropods may cause pain to mammals and this ability might shed light on the issue of arthropods feeling pain. Some use stings (e.g., scorpions, wasps, bees, and ants), some may use a proboscis (e.g., assassin bugs), and some may bite and inject venom (e.g., centipedes and spiders). Some may spray chemicals, as in bombardier beetles and ants, and some caterpillars have venomous spines. Most of the interest in these arthropods is about their effects on humans, who may suffer pain and possible death. However, these arthropods have a long evolutionary history, and the venoms may pre-date humans and most mammals. The venoms have evolved for two functions, that is, to kill prey and deter potential predators, and these are likely to include arthropods. They may also be used in intraspecific contests. Ants also use chemical sprays that deter other species of ants [92].

There is considerable variation in the venom within wasps and bees although there are some common components [93]. The differences in components often relate to the lifestyle of the species. For example, solitary wasps are predatory and use their stings to subdue, paralyze, or kill their prey. However, bees evolved a non-predatory life from wasp ancestors but retained the sting [94]. Here, the sting is often used to deter potential predators, particularly important in social species, and these venoms cause pain in mammals [93,95]. They also use stings, however, in defence against other invertebrates and in intraspecific contests. The question posed here is: Could the evolution of these mechanisms be driven (at least in part) by the benefits of inflicting pain on arthropods? As previously mentioned, the components of bee venom that cause pain in humans may induce autotomy in spiders, whereas those that do not cause human pain do not induce autotomy in spiders [67]. 

Scorpions use their stings in predation and defence, and they use the venom in a judicious manner [96]. They might use dry stings in defence when the risk is low, and when the risk increases, so does the volume of venom [96]. Further, scorpions may alter the composition of the venom and use components that lead to the death or subjugation of prey but select components that cause pain (at least in mammals) when trying to deter an attack by another animal [97]. 

Assassin bugs (*Pristhesancus plagipennis* and *Rhynocoris iracundus*) also have two main types of venom, one used in predation and another used in defence [98,99]. The predation venom causes paralysis and helps to break down the tissues of the prey to a liquid form. The defensive venom has components that may trigger nociceptors and might cause pain to deter a potential predator. Thus, the function of these components might be to cause pain to other arthropods. Insertion of the proboscis of assassin bugs causes pain in humans and autotomy in spiders. 

Spiders inject venom when they bite and it contains a substantial array of components [100]. Many of these activate nociceptors and result in pain (in humans) but there can also be antinociceptive compounds that have the opposite effect. The functional significance of these substances that influence nociceptors is not clear and it is not known if they are used to deter arthropod predators [100]. Most attention is directed toward their use against vertebrate predators, but it is estimated that the venoms were originally used against arthropods [101].

Bombardier beetles produce a noxious spray that is used to deter potential predators such as ants, spiders, or praying mantids, and the spray might also cause attacking spiders to autotomise legs or mantids to groom the body area hit by the spray [102,103]. The spray of bombardier beetles is the result of mixing two sets of chemicals ordinarily stored separately in the glands. One gland contains hydroquinones and hydrogen peroxide while the other contains catalases and peroxidases [104]. The beetle mixes the contents of the two compartments, causing oxygen to be liberated from hydrogen peroxide and the hydroquinones to be oxidized by the freed oxygen. The resulting liquid is released at about 100 °C and propelled at the attacker. Heat is a key component of the deterrent [104] and is likely to trigger nociceptors and possibly induce pain.

Some limacodid caterpillars possess spines that inject venom, and this protects against invertebrate predators [105]. Prior exposure to the venom also induces avoidance by arthropod predators, suggesting that it induces rapid avoidance learning. Some wasps manage to consume these caterpillars by first chewing off the venom spines, and assassin bugs pierce the caterpillar from the other side of the leaf [105]. The venom is painful to humans and has been linked to protection against vertebrate predators by inducing pain, however, they also protect against common arthropod predators.

It would be interesting to know more about the evolutionary history of these defensive venoms and if they induce signs of pain in other arthropods. It might inform us about the possible evolution of sentience in arthropods and how the ability to suffer from pain might be used to the advantage of other animals. It would also be useful to ask questions about pain in studies of intraspecific contests and traumatic mating.

## 6. Cognitive Ability and Possible Evolution of Pain in Arthropods

High cognitive ability has been suggested to be a prerequisite for pain [8,12,106,107,108,109,110,111]. High cognitive ability enables large amounts of sensory information to be integrated, enables effective decisions [112], and provides flexibility in the responses to noxious stimuli that are guided by some expectation of outcome. Recently, the ability to choose between possible responses to noxious stimuli, rather than relying on an inbuilt algorithm, has been suggested to lead to the ability to feel pain [25].

The ability to acquire and manipulate information has been shown in various arthropods. For example, hermit crabs gather information about potential new gastropod shells and integrate that with information about the shell they currently occupy before deciding which is the better of the two [34]. They use several sources of information such as the external and internal shape and size using visual and tactile information, and subsequently gather and integrate further information after moving in [113]. Hermits might also select a shell that would normally be avoided if that specific shell allows them to pass through a small hole to escape from a restrictive area, which suggests a degree of awareness of how to solve a problem [114]. Further, hermit crabs appear to be aware of experimentally induced changes to their shell, such as a plastic plate being attached, which impedes passage through small gaps [115]. The crabs seemed to assess the width of the gap and turn sufficiently to pass through. They quickly adapted to the attached plate and made greater turns to get through. 

The integration of information is even more complex when hermit crabs fight over the ownership of shells [90]. They gather information about both the shells and the opponent [116,117,118,119]. The attacker also monitors changes in its physiological state, which changes dramatically due to the exertions of the major fight activity of shell rapping [120,121], and shifts its fight behaviour according to those changes. Further, defenders assess the vigour of an attack and make decisions about whether to resist, the former involving mobilisation of glycogen stores [122]. They can also remember previous opponents for up to 4 days after an encounter [123]. Remarkably, a potential attacker might not only assess if it can gain from an exchange of shells but also assess if its shell is suitable for a potential opponent before attacking because the opponent might be more willing to exchange shells if it can gain in shell quality [124]. These crustaceans show an excellent ability to gather, manipulate, and use information from multiple sources, indicating a higher cognitive ability than generally recognised [90]. 

There are various examples of homing by crustaceans [125]. For example, fiddler crabs (*Uca* sp.) appear to show path integration by which turns are taken on the outward path during foraging, presumably by updating vectors and recalling them during the return [126]. There is also an impressive example of long-distance migration in the spiny lobster, *Panulirus argus,* during which they orientate by use of a magnetic sense [127]. Lobsters displaced by 12–37 km were capable of accurate orientation toward their home location. This necessitated a detection system to provide information about the current location and the home place location, coupled with a directional or compass sense to enable the home path to be determined [128].

Insects also show remarkable cognitive abilities and the best known of these are in Hymenoptera, especially bees [129]. It is possible that these abilities arose due to foraging on flowers and the need for minimising energy expenditure while maximising energy gain. The flowers of different species vary in many ways, including colour, odour, shape, ease of access to nectar, and nectar quality, and discriminating between flower types is key to the success of bees. They can learn to discriminate between two types of flowers and to switch preferences between contexts [129]. Bees can even learn concepts such as whether a visual stimulus is symmetrical or not. That is, after being exposed to several stimuli that differ in symmetry, and with either symmetrical or asymmetrical shapes being rewarding, the bee then discriminated entirely new stimuli based on their symmetry [130,131]. Social learning, in which one animal observes the actions of another and then employs those actions to obtain a reward, has also been noted in bumblebees. One experiment provided disks with food placed under a plexiglass sheet. The food could be accessed by pulling a string attached to the disk but very few bees learned this by themselves. However, those observing experienced bees pulling the string employed that method [132]. These examples, and many others, of high cognitive ability in bees have been reviewed by Chittka [129]. Together, they demonstrate remarkable cognitive abilities that go beyond previous expectations.

High cognitive ability has also been noted in spiders. Jumping spiders, for example, adjust their hunting methods depending on the type of prey [133]. The hunting spider (*Portia labiata*) hunts spitting spiders (*Scytodes pallidus*), which are also predators of spiders and thus dangerous. *Portia* gathers information as to whether the spitting spider is carrying eggs in its mouth and is thus less able to defend itself and modifies its attack accordingly [134]. Further, when hunting in complex environments, *Portia* plans the route to the prey and might take detours to avoid obstructions that initially take it away from the prey item [135]. This suggests an ability to comprehend the complex spatial relationships between itself and the prey and possible routes to a goal [136].

These examples of gathering and integrating sources of information that enable effective decisions demonstrate that various taxa of arthropods have the behavioural flexibility that has been suggested as a requirement for pain to be of use [25]. These observations on cognitive abilities add to the evidence concerning the behavioural criteria for pain in crustaceans, insects, and, to a lesser extent, spiders. This accumulation of evidence thus makes pain at least a possibility in arthropods. Next, it is considered how pain might have evolved in this phylum.

## 7. Evolution

If pain occurs in crustaceans, insects, and spiders, then the parsimonious explanation for the evolution of pain is that there was one evolutionary step that preceded the split between the Chelicerata and Mandibulata, which occurred in the Cambrian, over 500 million years ago [137]. However, there is less evidence of pain in spiders than in crustaceans and insects, and some might consider this insufficient to accept the possibility of pain in that group. If the spiders are thus disregarded, the parsimonious explanation for the evolution of pain is that it evolved before the crustacean/insect split (approximately 500 million years ago) [137]. However, within these groups, evidence for pain is found primarily in certain groups, such as the decapods in the crustaceans [37] and six orders of insects (Blattodea, Diptera, Hymenoptera, Lepidoptera, Orthoptera, and Coleoptera) [24]. A patchy distribution of pain might occur if pain was lost in some lineages. For example, taxa that evolved from a free-living form to a sedentary lifestyle may have reduced their behavioural choices and, thus, there may be no need for free choice and pain. For example, barnacles, especially those that have adopted a parasitic lifestyle, might have very little opportunity to show flexible behavioural responses to noxious stimuli and thus not benefit from a pain system [25]. However, a lack of flexibility might also be expected in basal groups of arthropods because of the limited ability of primitive sensory systems to gather information [25]. Improvements in sensory abilities and the associated cognitive abilities have been key features of animal evolution [138,139]. This suggests the ability to experience pain may have arisen multiple times, even within a single phylum such as Arthropoda [138,139]. If the above proposal is correct, then it might provide some guidance as to which species might experience pain due to their flexible decision-making following tissue damage [25]. Animals with a mobile predatory lifestyle require rapid decision-making and are thus expected to be the most likely candidates for sentience. These may be found among crustaceans, insects, centipedes, scorpions, and spiders, and these appear to be worthy of detailed investigations. 

## 8. Conclusions

There is no proof of pain in any animal, but it is possible to determine if pain is possible or even probable by determining if the expected criteria are fulfilled [12]. Here, there has been a focus on the behavioural criteria linked to the function of pain, i.e., changes that help the animal to subsequently avoid the conditions that produced the pain and changes that should enhance healing and survival. Such activities are found in crustaceans, insects, and, to a lesser extent, spiders. There are few such indicators in centipedes, millipedes, horseshoe crabs, and scorpions but these groups have received little attention regarding possible pain. 

Pain offers advantages beyond those gained from nociceptive reflexes [8,15] but there are costs. These involve the development of neural circuits that enable pain to function. Further costs may occur, however, if animals manipulate the pain systems of others for their own gain. For example, males might evolve genitals that damage the reproductive tract of females, possibly to dissuade the females from mating with another male [82]. Females may also dissuade males from prolonged copulation [86]. Further, animals engage in fights over resources, and it is possible that pain is inflicted to encourage the opponent to give up. It is known that animals trade-off the avoidance of noxious stimuli with competing motivations and this might provide the mechanism by which animals incur greater costs in fights for high-value resources [90,91]. Finally, many arthropods produce venom, especially venom that dissuades a potential predator (or competitor). These venoms are often painful to humans, but it seems likely that at least some components of defensive venoms arose to function against arthropods. That suggests they might be effective at triggering pain responses in other arthropods [105]. These situations of intraspecific and interspecific conflict may also provide insights into the evolution of pain. 

High cognitive ability has been suggested to be a requirement for pain [111]. Cognitive ability appears to increase as selection acts to improve the sensory systems and thus increase the information-gathering abilities of animals. The cognitive abilities of some arthropods are surprising and include the integration of information gained by diverse sensory systems and possible problem-solving in hermit crabs [90]. Also of note are concept formation [131] and social learning [129] in bees. This ability to integrate and manipulate information leads to a wider choice of behavioural responses to tissue damage and those choices might be based on some expectation of the utility of each candidate [25]. It has been argued that such an ability might result in the pain experience to guide and motivate the animal as it attempts to cope with tissue damage and make the best of a difficult situation [25]. There is evidence to suggest this may have occurred in some groups of arthropods, but probably not all.

## Figures and Tables

**Table 1:** The behavioural criteria for which there is evidence is shown for each taxon.

Behaviour	Mandibulata				Chelicerata		
	Crustacean	Insect	Centipede	Millipede	Spider	Scorpion	Horseshoe Crab
Avoidance	√	√			√	√	
Anxiety	√	√					
Long-term changes	√	√					
Trade-offs	√	√					
Directed activities	√	√					
Autotomy	√	√	√		√		

## Data Availability

Not applicable.
